# Kidney stone formation and antioxidant effects of *Cynodon dactylon* decoction in male Wistar rats

**Published:** 2017

**Authors:** Alireza Golshan, Parichehr Hayatdavoudi, Mousa AL-Reza Hadjzadeh, Abolfazl Khajavi Rad, Nema Mohamadian Roshan, Abbasali Abbasnezhad, Seyed Mojtaba Mousavi, Roghayeh Pakdel, Batool Zarei, Azita Aghaee

**Affiliations:** 1 *School of Medicine, North Khorasan University of Medical sciences, Bojnurd, Iran*; 2 *Neurocognitive Research Center, Department of Physiology, School of Medicine, Mashhad University of Medical Sciences, Mashhad, Iran*; 3 *Neurogenic Inflammation Research Center, Department of physiology, School of Medicine, Mashhad University of Medical Sciences, Mashhad, Iran *; 4 *Department of pathology, Ghaem hospital, Mashhad University of Medical Sciences, Mashhad, Iran*; 5 *Department of Physiology, School of Medicine, Gonabad University of Medical Sciences, Gonabad, Iran*; 6 *Department of physiology, School of Medicine, Mashhad University of Medical Sciences, Mashhad, Iran*; 7 *Pharmacological Research Center of Medicinal Plants, Department of Pharmacology, School of Medicine, Mashhad University of Medical Sciences, Mashhad, Iran*

**Keywords:** Cynodon dactylon decoction, FRAP, MDA, Nephrolithiasis, Antioxidant, Ethylene glycol

## Abstract

**Objectives:**

The antioxidant capacity impairs in kidney and urinary bladder of animals with stone disease. Herbal medicine can improve the antioxidant condition of renal tissue. *Cynodon dactylon* (*C. dactylon*) is a medicinal plant with antioxidative and diuretic properties and different preparations of this plant have shown promising effects in stone disease. Assessment of the whole plant decoction to prevent kidney stone disease as well as its antioxidant effects was the aim of this paper.

**Materials and Methods:**

Fifty male Wistar rats were randomly divided into 5 experimental groups (n=10). One group was left without treatment and four groups received ethylene glycol (1% v/v) in drinking water for 6 weeks. Three doses of *Cynodon dactylon* aqueous decoction (12.5, 50 and 200 mg/kg BW) were added to the drinking water of groups 3-5. Finally, water intake, 24-hour urine volume, MDA, total thiol concentration and FRAP value were measured in the serum and kidney tissues. The CaOx depositions were evaluated by hematoxylin and eosin staining.

**Results:**

Compared to the ethylene glycol-treated group, 200 mg/kg *C. dactylon*, lowered stone incidents, decreased urine volume, increased FRAP/g Cr (43%) and thiol content (p<0.05) with no significant alteration of water intake, MDA decreased significantly compared to *C. dactylon* 12.5 (p<0.01). Kidney weight increased and body weight decreased in ethylene glycol-treated group compared to the control group (p<0.05).

**Conclusion:**

A minimum dose of 200 mg/kg *C. dactylon* reduced stone formation and simultaneously increased total antioxidant power of serum and preserved MDA content and water.

## Introduction

Kidney stone, the third prevalent diseases of the kidney (Stoller and Bolton, 2004 ▶), is more frequent in males than females, with a ratio of 3:1 (Mckenzie and Hall, 2013 ▶). Its global incidence and predominance are also increasing with age in both sex groups (Romero et al., 2010 ▶), with about a 10% lifetime prevalence rate (Mckenzie and Hall, 2013 ▶). The recurrence rate of kidney stone in Iran, for example, is 32% after 5 years and 53% after 10 years (Safarinejad, 2007 ▶). Most of the renal calculi are made of calcium oxalate (60-70%) (Mckenzie and Hall, 2013 ▶). Administration of 0.75-1% of ethylene glycol can induce calcium oxalate (CaOx) nephrolithiasis within 4-6 weeks, in rats (Romero et al., 2010 ▶). Reactive oxygen species are among the most probable causes of stone formation (Khan, 2010 ▶), and treatment with vitamin E has prevented calcium oxalate deposition in the rat kidney (Itoh et al., 2005 ▶). Another form of treatment comes from medicinal plants such as *Nigella sativa* with well-known antioxidative effects (Hadjzadeh et al., 2011 ▶). *Cynodon dactylon* pers (*C. dactylon*), is a medicinal plant with antioxidant and diuretic effects (Khajavi- Rad et al., 2011 ▶). Different preparations of various parts of* C. dactylon* (roots, rhizomes and different fractions) have been shown to be effective in reducing renal calculi (Khajavi- Rad et al., 2011 ▶). Despite the effectiveness of the plant for this purpose, the mechanism of action has not been investigated. Usage of the whole plant decoction is more common and simpler than using specific parts, like rhizomes or roots. To our knowledge, the whole plant anti-lithiasis activity has not yet been investigated (Miraldi et al., 2001 ▶). The aqueous extract of aerial parts of *C. dactylon* has antioxidant properties (Devi et al., 2011 ▶; Khlifi et al., 2013 ▶; Shabi et al., 2010 ▶) and the rhizome’s extract has diuretic and anti-lithiasis activity (Atmani et al., 2009 ▶; Sadki et al., 2010 ▶). Furthermore, it is said that the aqueous extract of *C. dactylon *has higher antioxidant effects in comparison to the other preparations of the plant (Khlifi et al., 2013 ▶). So, in this study and for the first time, decoction of the whole plant (aerial parts, rhizomes and roots) was evaluated for its anti-lithiasis and anti-oxidant effects in reducing kidney stone formation in a rat model. 

## Materials and Methods

**Preparation of** ***Cynodon dactylon*** **Pers** **decoction**

*C. dactylon *pers is from the Poaceae family, and is known as Bermuda grass in English and Panjeh Morghi in Persian. The plant was freshly obtained from Imam Reza garden in Mashhad, Iran and its identity was verified by an expert botanist from the herbarium of the University of Ferdowsi, Mashhad, Iran (Voucher specimen no. 38314) after checking with* The Plant List* (www.theplantlist.org). The whole plant was desiccated in the shade, then ground to a soft powder. To make the decoction, 2 liters of distilled water was boiled then 200 g of the powder was added to the boiling water, and the mixture was allowed to boil for 2 hr, stirred every 15 min, and passed through progressively smaller sieves until finally passing through filter paper under vacuum conditions. The solution was then placed in a Rota vapor (Rotary evaporator, Laborota 4003, Heidolph Co. Germany) to remove the solvent under 45 ºC and 54 mm Hg. The prepared extract was transferred to a Petri dish and kept at room temperature until fully desiccated. It was kept in a sealed container in the refrigerator until ready for use; the extract yield was 15%. 

**Experimental design**

All procedures were approved by the Ethics Committee of Mashhad University of Medical Sciences, Mashhad, Iran in accordance with the Guide for care and use of laboratory animals, 8^th^ edition. In this study, 50 male Wistar rats (weighing 250-300 g) were purchased from the animal house of the School of Medicine, Mashhad University of Medical Sciences. They were kept in a 12-12 hr light-dark cycle at 22-25ºC and fed with a standard rat chow. The animals were randomly divided into 5 groups (n=10): 1- control (left without any treatment), 2- ethylene glycol group (1% v/v ethylene glycol (EG) (Merck, Darmstadt, Germany) in drinking water), and 3-5 treatment groups (*C. dactylon* extract 12.5, 50 and 200 mg/kg BW, respectively + 1% EG (v/v) in drinking water). The required amount of the extract was weighed from the concentrated stock sample and dissolved in distilled water to obtain a uniform solution. Following determination of daily water intake using a metabolic cage, the amount of the extract for each rat (mg/kg) was added to drinking water of treatment groups 3-5, i.e. 12.5, 50 and 200 mg/kg BW, according to the number of rats in each Plexiglas cage and their individual water needs (Hadjzadeh et al., 2007 ▶; Khajavi- Rad et al., 2011 ▶; Mohebbati et al., 2016 ▶; Shekha et al., 2014 ▶). A 24-hour urine sample, as well as water intake was individually measured in a metabolic cage at the beginning and at the end of study; blood samples were also collected from the retro-orbital sinus and the weight of the animals were recorded on days 0 and 42. The animals were treated for 6 weeks. At the end of the experiment, under ether anesthesia, a longitudinal abdominal incision made the kidneys visible and the renal arteries were clamped; the kidneys were then removed and animals sacrificed by deep ether anesthesia. The kidneys were washed with normal saline, weighed and placed in 10% formalin solution for further histological studies. Blood samples were centrifuged for 20 min at 3500 RPM and serum was kept at -20ºC until assay. 

**FRAP (Ferric reducing/ antioxidant power) Assay**

In the FRAP assay, the oxidized colorless form of Iron (Fe^³+^) is reduced to a blue colored Fe^²+^ - tripyridyltriazine compound through an electron donation by antioxidants. The total antioxidant power was evaluated, as described by (Hosseinzadeh et al., 2007 ▶). 

**Malondialdehyde measurement**

The common products of lipid peroxidation are malondialdehyde (MDA) and other aldehydes. They react with thiobarbituric acid (TBA), and the pink colored product has an absorbance at 532 nm. MDA was measured based on the Satoh method, and the molar extinction coefficient for MDA was 1.56 ×10^5 ^M^−¹ ^cm^−¹ ^(Satoh., 1978 ▶).

**Total sulfhydryl (thiol) groups measurement**

The thiol content was measured by Ellman or DTNB reagent (2, 2^ʹ^_ - _dinitro, 5, 5ʹ -dithiodibenzoic acid) that reacts with thiol groups to produce a yellow colored product with peak absorbance at 412 nm (Hosseinzadeh et al., 2005 ▶). 

**Histological studies**

Kidney tissues were washed with saline and put in 10% formalin then processed (Pro 200 tissue processor, Italy) and paraffin blocked. On each slide, 3 sections of 4μm were stained by Hematoxylin & Eosin. The renal tubules containing CaOx crystals were counted under light microscopy with magnification of ×400 in 10 microscopic fields. The results were reported by an expert pathologist who was blinded to treatment groups.

**Statistical analysis**

Data were analyzed using the Graph pad Prism 5 and presented as Mean ± SEM with the following tests: the one way analysis of variance (ANOVA), Kruskal-Wallis (to compare all groups with unequal variances) followed by *post-hoc* Dunn’s test, two way ANOVA, paired and unpaired t test. A p<0.05 was considered significant. 

## Results

**Figure 1:**
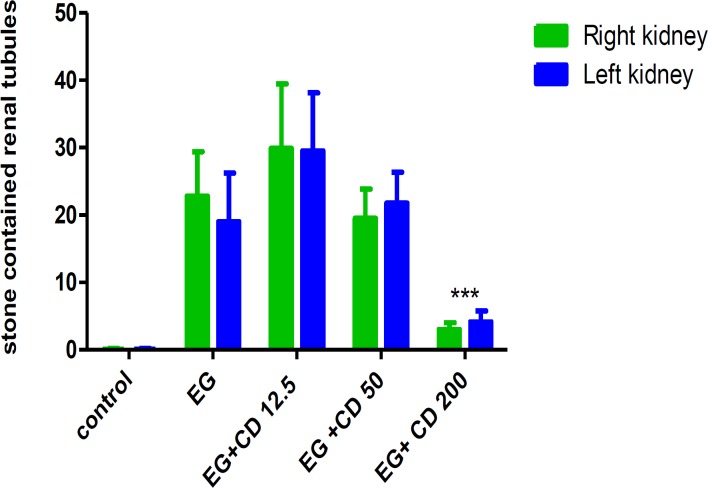
The number of stones in the right *vs* left kidney in the control, ethylene glycol and *C. dactylon*-treated male Wistar rats. Data were analyzed by two way ANOVA. *** p<0.001 compared to *EG*. EG: Ethylene glycol, CD: *C. dactylon*

*C. dactylon *had a significant effect on the rate of stone occurrence (p<0.001). There was not a statistically significant difference for stone formation in the right *vs* left kidneys (Figure 1), laterality and interaction were not significant.

**Figure 2:**
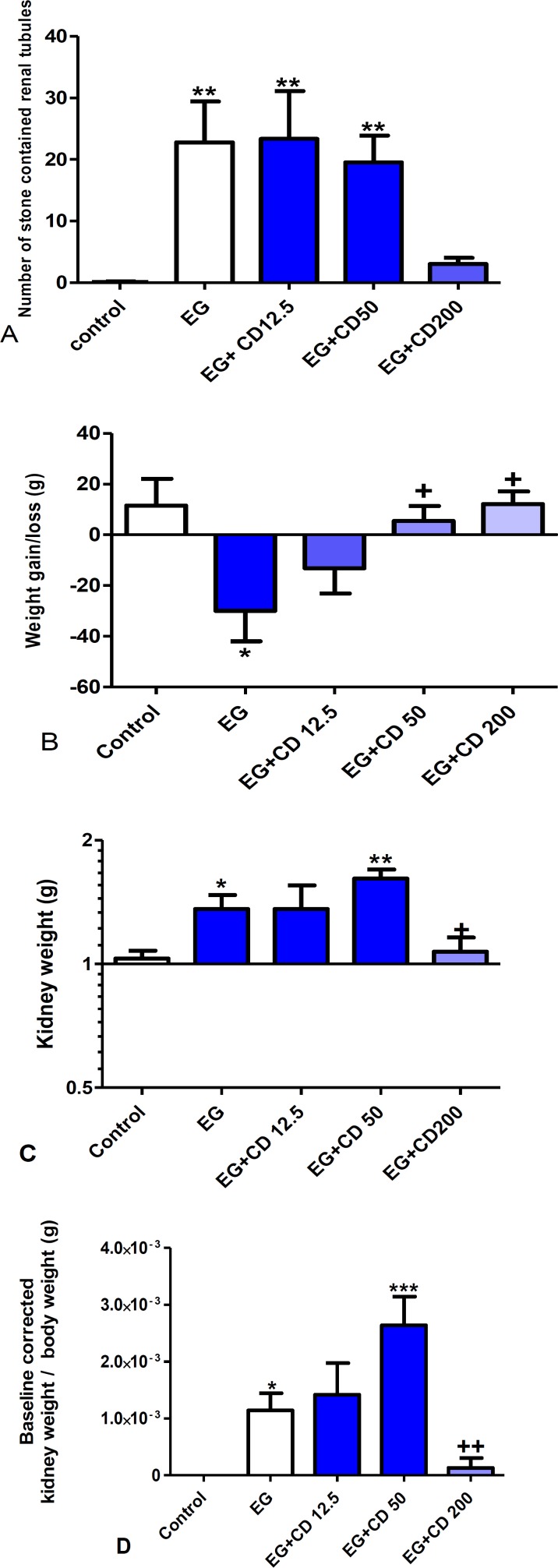
(A) The occurrence of renal calculi in the control, ethylene glycol and *C. dactylon*-treated male Wistar rats. (B) Weight gain/loss. (C) Kidney weight. (D) Kidney weight to total body weight ratio (baseline corrected to the control group). * p<0.05, ** p<0.01 and *** p<0.001 compared to the control group, + p<0.05 and ++ p<0.01 compared to EG*+CD 50*. Data of renal calculi was analyzed by Kruskal Wallis followed by Dunn’s test and data of weight by one way ANOVA followed by Newman-Keuls multiple comparison test. EG: Ethylene glycol, CD: *C. dactylon*

There was also a significant correlation between the *C. dactylon *dose and renal stones, Pearson r= -0.9701, R^2^=0.94, p=0.029. The number of tubules containing CaOx crystals increased significantly in the ethylene glycol, *C. dactylon *12.5 and 50 mg/kg (p<0.01) compared to the control group (Figure 2A). The dose of 50 mg/kg of *C. dactylon* showed less, however, big stones than *C. dactylon *12.5 mg/kg (Figure 2B). Apparently, *C. dactylon* 200 mg/kg , prevented from a significant crystal formation. There was a significant weight loss in ethylene glycol group compared to the control group, p<0.05. However, 50 and 200 mg/kg of* C. dactylon* not only prevented the EG-induced weight loss but also induced a significant weight gain compared to EG group (p<0.05) (Figure 2B). Rats treated with *C. dactylon* and ethylene glycol showed a significant increment in urine volume in all treated groups except in *C. dactylon* 200 mg/kg, compared to the control group (p<0.01). 

**Figure 3:**
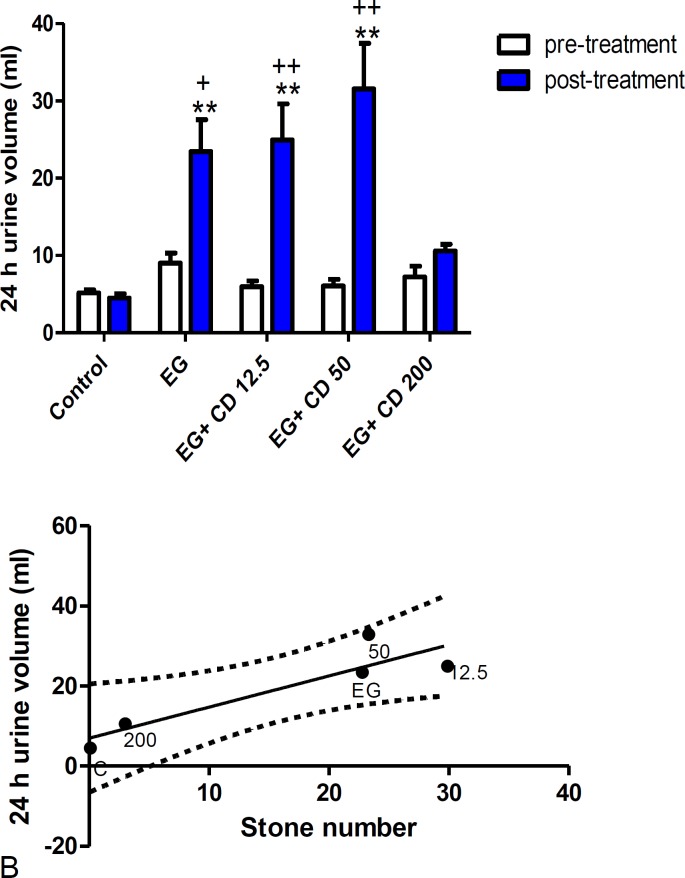
). The comparison of 24-hr urine volume in the control, ethylene glycol and* C. dactylon-*treated male Wistar rats. ** p<0.01 compared to the control group, + p<0.05 and ++ p<0.01 comparison between pre and post-treatment groups. Data analysis was done by Kruskal-Wallis followed by Dunn’s test to reveal statistical difference among groups and paired t test for comparisons within each group (pre and post-treatment groups). B) Linear regression analysis showing the relationship between the stone number and the 24-hr urine volume, 95% upper and lower CI. EG: Ethylene glycol, CD: *C. dactylon*

Also, paired t test revealed a significant urine volume increase in the groups of ethylene glycol (p<0.05), *C. dactylon* 12.5 and 50 mg/kg (p<0.01) but not in *C. dactylon* 200 mg/kg. No significant difference was observed among all groups compared to the ethylene glycol group after different treatments; although, *C. dactylon* 200 mg/kg reduced urine volume about 54% compared to the ethylene glycol group (Figure 3A).

There was a significant increase in water intake in the groups of *C. dactylon *12.5 and 50 mg/kg (p<0.01) compared to pre-treatment phase and also in the same groups compared to the control group, *C. dactylon* 12.5 (p<0.05) and 50 mg/kg (p<0.01). Furthermore, there was a positive correlation between daily water intake and stone occurrence (r²= 0.83) and between the urine volume and stone occurrence (r²=0.81). 

**Figure 4:**
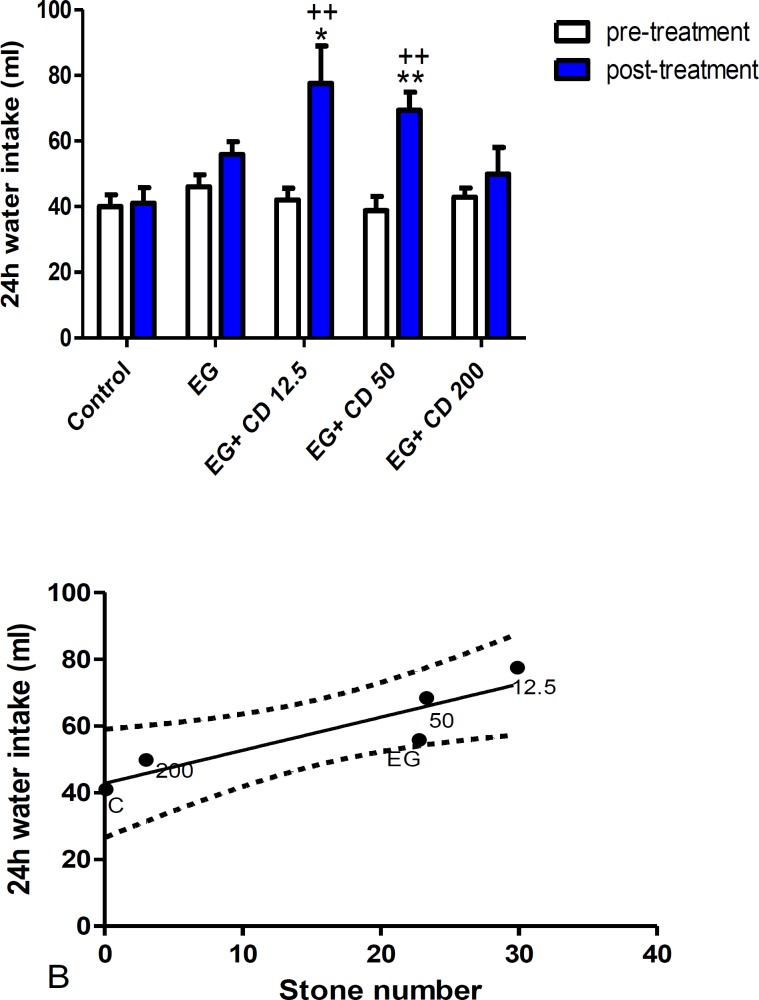
A) The comparison of daily water intake in the control, ethylene glycol and *C. dactylon*-treated male Wistar rats. *p<0.05 and **p< 0.01 compared to the control group. ++ p<0.01 comparison between pre and post-treatment groups. Data analysis was done by Kruskal-Wallis test followed by Dunn’s test to reveal statistical difference among groups and paired t test was done for comparisons within groups. B) Linear regression analysis showing the relationship between the stone number and the 24-hr water intake, 95% upper and lower CI. EG: Ethylene glycol, CD: *C. dactylon*

**Figure 5:**
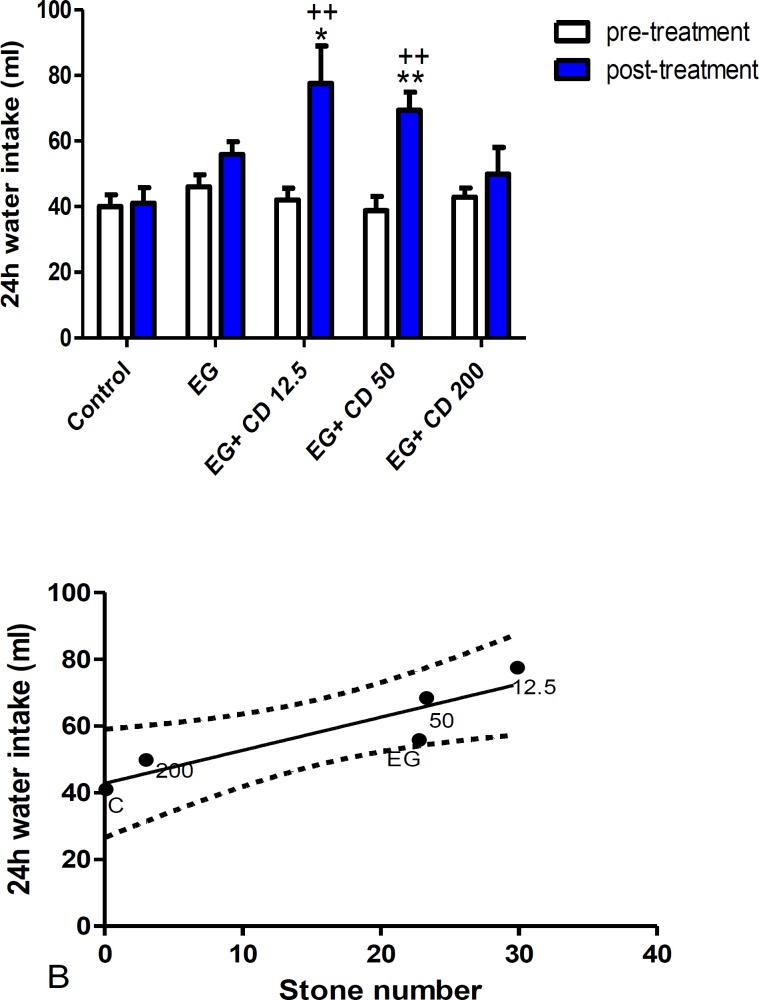
The results of the FRAP assay (plasma and kidney homogenates) and serum creatinin in the control, ethylene glycol and *C. dactylon*-treated male Wistar rats. A) Serum FRAP values, B) Serum FRAP value/g Cr before and after treatments. C) kidney homogenate FRAP values. D) Serum creatinin. +p<0.05, ++ p<0.01 and +++p<0.001 compared to the control group. * p<0.05 and **p<0.01 compared to pre-treatment phase. Serum FRAP values analyzed by Kruskal test followed by Dunn's test and serum creatinin was analysis by one way ANOVA followed by Bonferroni *post-hoc* test. EG: Ethylene glycol, CD: *C. dactylon*

A significant increase in serum FRAP value was observed in groups of *C. dactylon *12.5 (p<0.01), 50 (p<0.05) and 200 mg/kg (p<0.001) compared to the control group, and also between pre and post-treatment phases within the groups of ethylene glycol (p<0.01), 12.5 and 50 (p<0.05) (Figure 5A). Furthermore, *C. dactylon* 200 mg/kg induced an insignificant increase (43%) in FRAP/g Cr compared to the ethylene glycol group (Figure 5B). Despite a stepwise dose dependent increment of FRAP values in *C.dactylon* treated kidney homogenates, no significant change was evident in any groups (Figure 5C). Renal tissue FRAP value demonstrated 34.6%, 26% and 18.6% reduction compared to the control group, in groups of *C. dactylon* 12.5, 50 and 200 mg/kg, respectively. There was not a significant difference in serum Cr among groups after receiving different treatments (Figure 5D). 

**Figure 6:**
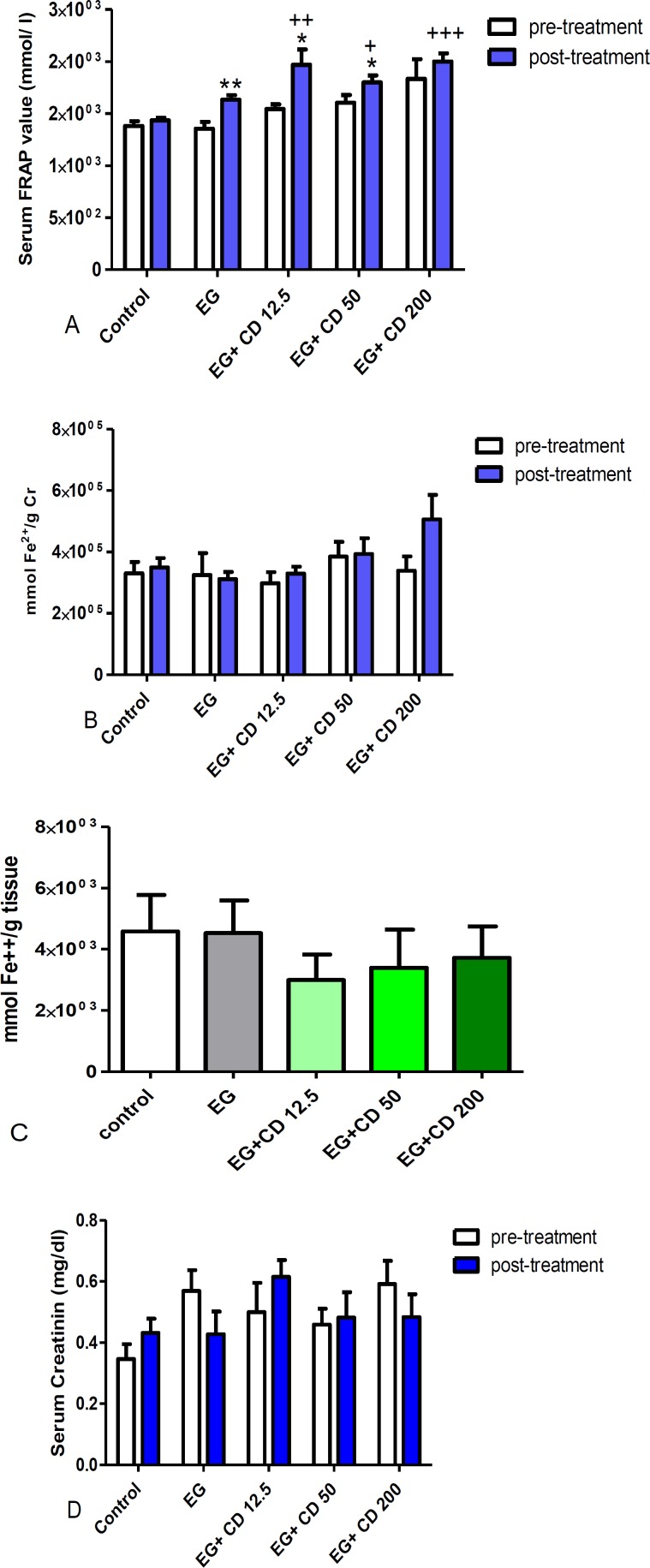
The total thiol (A) and MDA (B) content of the kidney tissues in the control, ethylene glycol and *C. dactylon*-treated male Wistar rats. (A) * p<0.05 and **p<0.01 compared to the control group, + p<0.05 and ++ p<0.01 compared to the ethylene glycol group, & p<0.05 *EG+CD 50* compared to *EG+CD12.5*. One way ANOVA followed by Newman-Keuls tests. EG: Ethylene glycol, CD: *Cynodon dactylon*

There was a significant reduction of the total thiol content in the ethylene glycol group (p<0.01) and *EG+CD12.5* (p<0.05) compared to the control group, however, the increased thiol content was noted in the groups of *EG+CD 50* and 200 compared to ethylene glycol group (p<0.01 and p<0.05, respectively) and *EG+CD12.5* (p<0.05). MDA concentration was transformed to -1*(log y) then a significant increase in MDA level was noted in *EG+CD12.5 *compared to the control group (p<0.01) and a significant reduction of MDA in the group of *EG+CD 200* compared to *EG+CD 12.5* (p<0.05). There is also a negative correlation between the log of the MDA level and the reciprocal of stone number in the control group (p=0.020, r²= -0.715). The kidney FRAP and thiol content revealed a positive correlation only in the group of EG+*CD* 200 (p=0.030, r²=0.717). A positive correlation is also present for the log of kidney FRAP and the reciprocal of stone number in the groups of *EG+CD 12.5* (p=0.016, r²= 0.805) and *EG+CD 200* (p=0.029, r²= 0.805).

**Figure 7:**
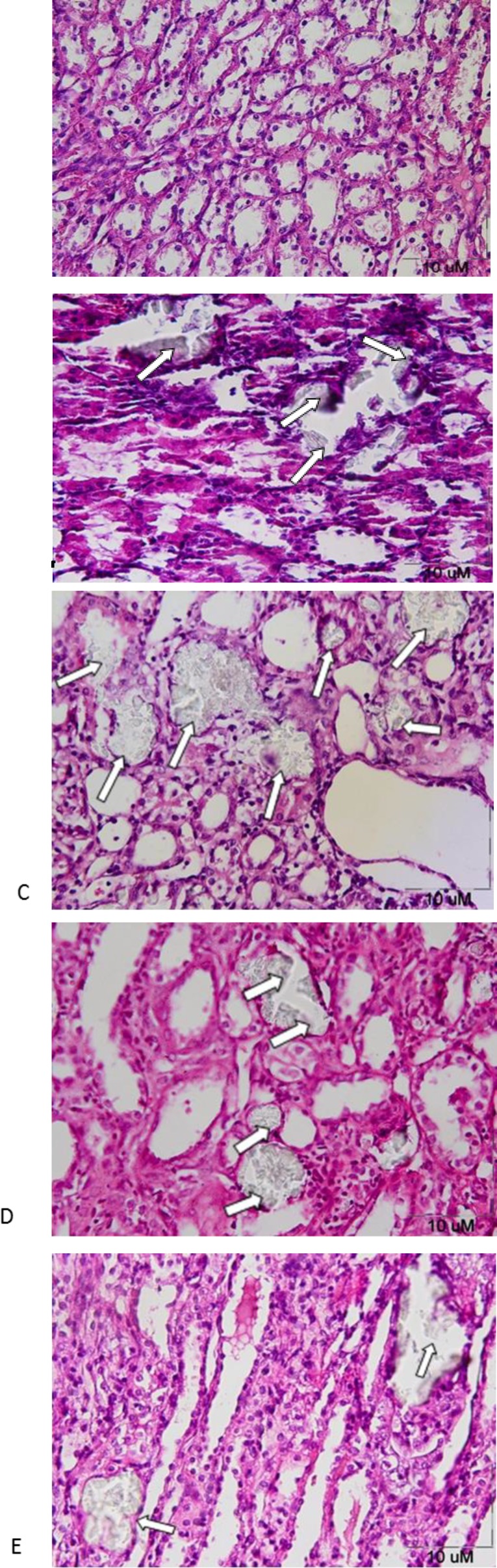
CaOx deposition in the control, ethylene glycol and *C. dactylon*-treated male Wistar rats. A) No significant pathologic finding was seen in the control group.  B) Intratubular crystals were found in the ethylene glycol group. C) Severe intratubular crystals with mild interstitial inflammation occurred in the *EG+CD 12.5* D) Intratubular crystals with mild interstitial inflammation occurred in the *EG+CD 50*. E) Intratubular crystals with mild interstitial inflammation in the *EG+CD 200* (only in two cases out of 9) (HE staining, 400X

No pathologic finding was observed in the kidneys of the control group (Figure 7A), but the CaOx crystals damaged the tubular walls in the ethylene glycol group; in this group, the kidneys represented a granular view, and in some cases, the medulla was destroyed. These were in accordance with the polyuria and very light yellow color urine samples obtained from this group (Fig 7B). The crystal accumulation in the group of *EG+CD 12.5* was more marked than all groups, and the widening of tubular lumen was seen; in one rat, the hematuria was observed and in another, the granular appearance was noted in addition to polyuria and polydipsia (Fig 7C). The kidneys of the *EG+CD 50* almost appeared normal and the crystal accumulation was less than the group of 12.5 mg/kg, also in one case light brown urine was noted (Fig 7D). In the *EG+CD 200 *group, tubules containing stones were considerably lower than other groups. Three animals had clouded and light brown urine; the integrity of the tissue was more preserved than other groups (Figure 7E).

## Discussion

Nephrolithiasis manifests many aspects eof arteriosclerosis including calcification, which in turn can be inhibited by antioxidants (Itoh et al., 2005 ▶). All antioxidant enzymes are decreased in kidneys with stone disease, except catalase. In patients with stone disease, hyperoxaluria leads to intracellular erythrocyte oxidative stress and ultimately, tubulointerstitial hypoxia impairs the ability of tubular cells to convey antioxidative enzymes, leading to more vulnerability to stone formation (Ma et al., 2014 ▶). Consistent with the above-mentioned studies, the current results indicate a significant reduction in the kidney thiol content in the ethylene glycol group while FRAP values did not change significantly and MDA level slightly increased above the level of the control group. It seems that the endogenous mechanisms strived to achieve the desired level of antioxidants and combat oxidative stress induced by ethylene glycol. Eukaryotic cells possess an internal free radical scavenging system to combat extra oxidant stress (Huang et al., 2002 ▶). The FRAP assay is an easy technique to evaluate the total antioxidant status of biological samples (Gupta et al., 2009 ▶), however, it does not evaluate thiol groups (Lim and Lim, 2013 ▶). Furthermore, it has been reported that an oxidant challenge did not alter the MDA level and had little effect on thiols (Giannerini et al., 2001 ▶). Recently, it has been reported that establishment of disulfide bonds requires enough oxidative redox potential, it is likely that such mechanism in part plays a role in the kidney stone disease (Watson, 2014 ▶) . 

Medicinal plants with antioxidative properties such as *Nigella sativa* showed preventive effects on kidney stones (Hadjzadeh et al., 2011 ▶, 2007). Furthermore, different fractions and parts of *C. dactylon* have been shown to be effective in the treatment of renal stones (Atmani et al., 2009 ▶; Khajavi-Rad et al., 2011 ▶). The aqueous extract of *C. dactylon* has been reported to have the lowest yield, the highest phenolic content and higher antioxidant activity than its other forms of extracts, *in vitro* (Khlifi et al., 2013 ▶). However, Arumugam et al. indicated that a hot water extract of *C. dactylon* has lower levels of phenolic content and the most phenolic content presented in the ethanolic form (Arumugam et al., 2014 ▶). These controversies may be due to the method of preparation, using different plat parts or different analytical assays. Consistent with this observation, the *C. dactylon *ethanolic extract, at low doses, has shown positive effects in prevention of kidney stones (Khajavi- Rad et al., 2011 ▶) which, may be due to a higher phytochemical constituents in the ethanolic extract (Shabi et al., 2010 ▶). Besides, *C. dactylon *has shown immunomodulatory and free radical scavenging effects in its fresh liquid form (Mangathayaru et al., 2009 ▶), in addition to anti-inflammatory and antioxidative properties in rats (Sindhu et al., 2009 ▶). The most potent antioxidant activity of the aqueous extract of *C. dactylon* (whole plant) attributed to the presence of newly-identified anthocyanins (Khlifi et al., 2013 ▶). Therefore, beneficial effect of the aqueous extract in this experiment might be due to the presence of anthocyanins.

Furthermore, it has been reported that the reactive oxygen species (ROS) significantly increased one week after ethylene glycol treatment in rats; however, they returned to baseline levels on days 21 and 42. Therefore, different mechanisms are responsible for ROS enhancement in the early and late stages of nephrolithiasis (Huang et al., 2002 ▶). This is in line with the results of the current study; because, following 6 weeks of treatment, MDA was almost at the same level of control group in EG, *C. dactylon *50 and 200 mg/kg and thiol content was close to control values in *C. dactylon *50 and 200 mg/kg. On the other hand, the ethylene glycol group has almost the same FRAP level of the control group and there was an insignificant reduction in kidney FRAP values in parallel to the stone occurrence with different *C. dactylon *doses, it might be concluded that mechanisms other than antioxidative property are also involved to prevent the stone formation by *C. dactylon*. 

The *C. dactylon *decoction not only prevented the EG- induced weight loss but also *C. dactylon *50 and 200 mg/kg induced a significant weight gain. The animals were calm during the experiment and no sign of pain was observed even in those with the high incidence of calculi. Increased kidney weight in EG group was in parallel with other studies (Alex et al., 2013 ▶; Shekha et al., 2014 ▶). 

 Higher stone incidents accompanied with higher urine volume and consequently higher water intake in this study. Increased medullary osmolality predisposes the kidney to oliguria and the risk of forming renal stones, and to avoid this, the kidney tends to increase the flow rate and conserving water (Halperin et al., 2008 ▶). Low urine volume is in favor of lithogenesis, therefore, increasing the urine volume will reduce the transit time of intratubular fluids and combat the formation of Randall’s plaques (Nouvenne et al., 2008 ▶; Ramegowda et al., 2007 ▶). Also, increased urine volume is a mechanism of action of herbal medicines to help the passage of stones from the kidneys (Yadav et al., 2011 ▶). The *C. dactylon *rhizome juice takes four hours to stimulate diuretic activity in rats and the diuresis persists for 24 hours after administration of a single dose (Sadkia et al., 2010 ▶). The diuretic effect may be due to the steroid saponins (Garjani et al., 2009 ▶) or the high mannitol content (Garjani et al., 2009 ▶) and flavonoids in this herb (Khajavi- Rad et al., 2011 ▶). In this study, the increase in urine volume was evident in groups with more stones. It seemed that the stones directed the kidney to increase the flow rate; if it was because of the extract then the highest dose (*C. dactylon *200 mg/kg) should represent the highest urine volume. Presumably, the occlusion of kidney tubules by stones has predisposed the kidney to renal failure and resulted in post-obstructive diuresis (Harris et al., 1975 ▶). 

There are inorganic urinary inhibitors, such as citrate, pyrophosphate and magnesium and organic stone inhibitors such as nephrocalcin, crystal matrix proteins like urinary prothrombin fragment 1 (UPTF1) and glycosaminoglycans (urinary poly-anions), which prevent CaOx aggregation (Bihl and Meyers, 2001 ▶; Ramegowda et al., 2007 ▶). Previous data of our laboratory indicated the effects of the *C. dactylon *extract on the inorganic compounds, such as lowering urinary citrate excretion and the reduction of serum magnesium in the treated rats (Khajavi- Rad et al., 2011 ▶); however, Atmani et al. reported no significant biochemical change except in the oxalate level in the preventive group and calcium, sodium and potassium change in the curative group treated with *C. dactylon *(Atmani et al., 2009 ▶). Whether the *C. dactylon *extract can positively affect the organic materials can be a new area of investigation. 

This is for the first time that antioxidant effects of the *C. dactylon *have been investigated in the context of renal stone. 200 mg/kg BW of *C. dactylon *extract reduced the stone incidents, at least in part through increasing the total antioxidant capacity, while preserving the MDA level; however, the exact mechanism of action and the main components responsible for this effect require more investigation.

## References

[B1] Alex M, Varghese MV, Abhilash M, Paul MVS (2013). Effect of astaxanthin on ethylene glycol induced nephrolithiasis. IOSR-JPBS.

[B2] Arumugam N, Boobalan T, Rajeswari PR, Duraimurugan MD (2014). Antimicrobial activity and phytochemical screening of Cynodon dactylon and Carica papaya. Res Biotechnol.

[B3] Atmani F, Sadki C, Aziz M, Mimouni M, Hacht B (2009). Cynodon dactylon extract as a preventive and curative agent in experimentally induced nephrolithiasis. Urol Res.

[B4] Bihl G, Meyers A (2001). Recurrent renal stone disease — advances in pathogenesis and clinical management. Lancet.

[B5] Devi KMS, Annapoorani S, Ashokkumar K (2011). Hepatic antioxidative potential of ethyl acetate fraction of Cynodon dactylon in Balb / c mice. J Med Plants Res.

[B6] Garjani A, Afrooziyan A, Nazemiyeh H, Najafi M, Kharazmkia A, Maleki-dizaji N (2009). Protective effects of hydroalcoholic extract from rhizomes of Cynodon dactylon ( L) pers on compensated right heart failure in rats. BMC Complement Altern Med.

[B7] Giannerini F, Giustarini D, Lusini L, Rossi R, Simplicio P Di (2001). Responses of thiols to an oxidant challenge : differences between blood and tissues in the rat. Chem Biol Interact.

[B8] Gupta R, Sharma M, Lakshmy R (2009). Improved method of total antioxidant assay. Indian J Biochem Biophys.

[B9] Hadjzadeh M, Khajavi-rad A, Rajaei Z, Tehranipour M, Monavar N (2011). The preventive effect of N-butanol fraction of Nigella sativa on ethylene glycol-induced kidney calculi in rats. Pharmacogn Mag.

[B10] Hadjzadeh MA, Khoei A, Hadjzadeh Z (2007). Ethanolic extract of Nigella Sativa L seeds on ethylene glycol-induced kidney calculi in rats. Urol J.

[B11] Halperin ML, Kamel KS, Oh MS (2008). Mechanisms to concentrate the urine : an opinion. Curr Opin Nephrol Hypertens.

[B12] Harris RH, Yarger WE, Carolina N (1975). The pathogenesis of post-obstructive diuresis: the role of circulating natriuretic and diuretic factors , including urea. J Clin Invest.

[B13] Hosseinzadeh H, Hosseini A, Nassiri-asl M, Sadeghnia HR (2007). Effect of Salvia leriifolia benth root extracts on ischemia-reperfusion in rat skeletal muscle. BMC Complement Altern Med.

[B14] Hosseinzadeh H, Sadeghnia HR, Ziaee T, Danaee A (2005). Protective effect of aqueous saffron extract ( Crocus sativus L) and crocin , its active constituent , on renal ischemia-reperfusion-induced oxidative damage in rats. J Pharm Pharm Sci.

[B15] Huang H, Ma M, Chen J, Chen C (2002). Changes in the oxidant-antioxidant balance in the kidney of rats with nephrolithiasis induced by ethylene glycol. J Urol.

[B16] Itoh Y, Yasui T, Okada A, Tozawa K, Hayashi Y, Kohri K (2005). Preventive effects of green tea on renal stone formation and the role of oxidative stress in nephrolithiasis. J Urol.

[B17] Khajavi- Rad A, Hajzadeh M, Rajaei Z, Sadeghian MH (2011). Preventive effect of Cynodon dactylon against ethylene glycol-induced nephrolithiasis in male rats. Avicenna J Phytomedicine.

[B18] Khajavi-Rad A, Hadjzadeh M, Rajaei Z, Mohammadian N, Valiollahi S, Sonei M (2011). The Beneficial effect of Cynodon Dactylon fractions on ethylene glycol-induced kidney calculi in rats. Urol J.

[B19] Khan SR (2010). Nephrocalcinosis in animal models with and without stones. Urol Res.

[B20] Khlifi D, Akrem E, Valentin A, Cazaux S, Moukarzel B, Hamdi M, Bouajila J (2013). LC – MS analysis , anticancer , antioxidant and antimalarial activities of Cynodon dactylon L extracts. Ind Crop Prod.

[B21] Lim CSH, Lim SL (2013). Ferric reducing capacity versus ferric reducing antioxidant power for measuring total antioxidant capacity. Lab Med.

[B22] Ma M, Chen Y, Huang H (2014). Erythrocyte oxidative stress in patients with calcium oxalate stones correlates with stone size and renal tubular damage. URL.

[B23] Mangathayaru K, Umadevi M, Reddy CU (2009). Evaluation of the immunomodulatory and DNA protective activities of the shoots of Cynodon dactylon. J Ethnopharmacol.

[B24] Mckenzie G, Hall J (2013). Management of stone disease. Surgery.

[B25] Miraldi E, Ferri S, Mostaghimi V (2001). Botanical drugs and preparations in the traditional medicine of west Azerbaijan ( Iran ). J Ethnopharmacol.

[B26] Mohebbati R, Shafei MN, Soukhtanloo M, Karimi S, Beheshti F (2016). Adriamycin-induced oxidative stress is prevented by mixed hydro- alcoholic extract of Nigella sativa and Curcuma longa in rat kidney. Avicenna J phytomedicine.

[B27] NouvenneA, Meschi T, Guerra A, Allegri F, Prati B, Borghi L (2008). Dietary treatment of nephrolithiasis. Clin Cases Miner Bone Metab.

[B28] Ramegowda D, Shekhar C, Browning AJ, Cartledge JJ (2007). The role of urinary kidney stone inhibitors and promoters in the pathogenesis of calcium containing renal stones. EAU-EBU Updat Ser.

[B29] RomeroV, Akpinar H, Assimos DG (2010). Kidney stones : a global picture of prevalence , incidence , and associated risk factors. Rev Urol.

[B30] Sadkia C, Hachtb B, Soulimana A, Atmania F (2010). Acute diuretic activity of aqueous Erica multiflora flowers and Cynodon dactylon rhizomes extracts in rats. J Ethnopharmacol.

[B31] Safarinejad MR (2007). Adult urolithiasis in a population-based study in Iran : prevalence , incidence , and associated risk factors. Urol Res.

[B32] Satoh KEI (1978). Serum lipid peroxide in cerebrovascular diorders determined by a new colorimetric method. Clin Chim Acta.

[B33] Shabi MM, Gayathri K, Venkatalakshmi R, Sasikala C (2010). Chemical constituents of hydro alcoholic extract and phenolic fraction of Cynodon dactylon. Int J ChemTech Res.

[B34] Shekha MS, Qadir AB, Ali HH, Selim XE (2014). Effect of Fenugreek (Trigonella foenum-graecum) on ethylene glycol induced kidney stone in rats. Jordan J Biol Sci.

[B35] Sindhu G, Ratheesh M, Shyni GL, Helen A (2009). Inhibitory effects of Cynodon dactylon L on inflammation and oxidative stress in adjuvant treated rats. Immunopharmacol Immunotoxicol.

[B36] Stoller M, Bolton D (2004). Smith’s general urology.

[B37] Watson JD (2014). Type 2 diabetes as a redox disease. Lancet.

[B38] Yadav RD, Jain SK, Alok S, Mahor A, Bharti JP, Jaiswal M (2011). Herbal plants used in the treatment of urolithiasis : a review. IJPSR.

